# Targeting CD47 as a therapeutic strategy: A common bridge in the therapy of COVID-19-related cancers

**DOI:** 10.1016/j.heliyon.2023.e17959

**Published:** 2023-07-04

**Authors:** Milad Zandi, Maryam Shafaati, Mohammad Shenagari, Hamed Naziri

**Affiliations:** a Department of Virology, School of Public Health, Tehran University of Medical Sciences, Tehran, Iran; b Department of Microbiology, Faculty Science, Jahrom Branch, Islamic Azad University, Jahrom, Iran; c Department of Microbiology, Faculty of Medicine, Guilan University of Medical Sciences, Rasht, Iran

**Keywords:** CD47, SIRPα, Phagocytosis, Innate immunotherapy

## Abstract

Macrophages are essential mediators of innate immunity. Non-self-cells resist phagocytosis through the expression of the checkpoint molecule CD47. CD47, as the integrin-associated protein, is overexpressed on tumor and SARS-CoV-2-infected cells as a potential surface biomarker for immune surveillance evasion. CD47-signal-regulating protein alpha (SIRPα) interaction is a promising innate immunotarget. Previous findings based on monoclonal antibodies (mAbs) or fusion proteins that block CD47 or SIRPα have been developed in cancer research. While CD47 efficacy in infectious diseases, especially severe COVID-19 studies, is lacking, focus on macrophage-mediated immunotherapy that increases “eat me” signals in combination therapy with mAbs is optimistic. This integrin-related protein can be as a potential target to therapy for COVID-19. Here, we concentrate on the role of the CD47 signaling pathway as a novel therapeutic strategy for COVID-19-associated cancer treatment.

## 1 Introduction

The unpredictability of infection outcomes is a major problem in the COVID -19 era due to coevolution of high-risk vaccine resistance variants. However, only a small percentage of individuals with severe COVID-19 develop an uncontrolled immune response leading to severe inflammation. In high-risk patients, antiviral intervention in the early stages of the disease may prevent progression to severe stages. Therefore, a better understanding of the underlying processes is needed to identify patients who may develop severe disease as early as possible [1]. Understanding immune escape processes will lead to the identification of biomarkers that predict disease outcome [[2], [3], [4]]. Currently, however, antiviral immunomodulatory agents are scarce. Because CD47 acts as an immune escape mediator in cancer and SARS-CoV-2-infected cells, it can be used as a new early antiviral therapy to boost antiviral immunity. This integrin-associated protein may be a potential target for COVID-19 therapy. To date, there have been few studies on the therapeutic role of CD47 in viral diseases. Given the role of CD47 in tumor cell immune escape, we focused on understanding the immunotherapeutic role of CD47 in the context of cancer and COVID-19 research and how the molecular pathway in cancer might affect SARS-CoV-2 infection and vice versa.

### 1.1 CD47 and immune responses

CD47 is a novel immune checkpoint of macrophage that plays an important role in immune evasion of the lethal duo, cancer, and severe COVID-19 [5]. Macrophages use CD47 expression to distinguish between “self” and “non-self” [6]. CD47 surface expression in all immune cells, including cancer cells, can be upregulated during infection. In severe COVID-19-associated cancer, CD47 is overexpressed. As a self-marker, this cell surface glycoprotein transmembranously interacts with signal regulatory proteins (signal regulatory protein α (SIRP) or CD172a) expressed on Macrophages (MQ), Dendritic cells (DCs) [7], and Cytolytic T lymphocytes (CTL) [8]. This interaction between MQ and DCs results in the activation of an anti-phagocytic signaling pathway known as “do not eat me” that causes virus-infected or tumor cells to largely to escape phagocytosis by MQ. Tyrosine phosphatases (Src homology 2 domain-containing protein tyrosine phosphatase 1/2 (SHP1/2)) recruit and activate phosphorylation of the cytoplasmic immunoreceptor tyrosine-based inhibitory motif (ITIM motif) after CD47-SIRP interaction in phagocytes. Therefore, cytoplasmic myosin-IIA expression was suppressed. In the presence of CD47, the anti-macrophage receptor and phosphatase activator SIRPα localizes to the synapse and prevents the synthesis of phosphotyrosine and myosin-II without affecting F-actin. Ultimately, both human CD47 and direct myosin inhibition significantly reduce phagocytosis [9]. Whether a macrophage consumes a target cell or particle ultimately depends on the factors that encourage cytoskeletal phagocytosis. This promotes the aggregation of several phagocytic synapse proteins, including uptake-assisting non-muscle myosin-II motors [10].

The immunomodulatory phosphatase Src homology region 2 domain-containing phosphatase-1 (SHP-1) is activated when CD47 attaches to the macrophage inhibitory receptor signal-regulating protein alpha (SIRPα), which controls a number of proteins, including non-muscle myosin-IIA.

**Fig. 1:**
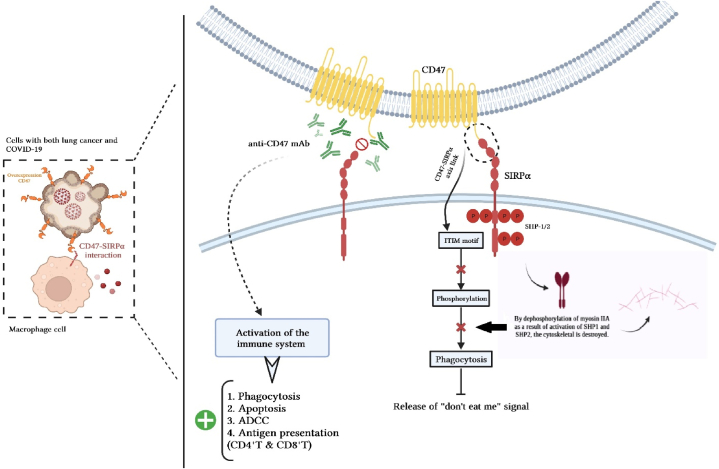
CD47, the link between cancer & COVID-19. The engagement of CD47-SIRPα plays an essential role in inhibiting the phagocytosis process. The interaction of CD47-SIRPα with phosphorylation of SHP1/2 in the ITIM motif triggers a series of events in the form of a signaling cascade to cause dephosphorylation of myosin IIA, which is an important stage in the phagocytic activity of macrophages. CD47-SIRPα interaction is associated with increased CD47 expression in tumor or SARS-CoV-2-infected cells. Immunotherapy by innate immune cells, including macrophages, is a new therapeutic perspective in understanding the common molecular pathways of cancer and COVID-19. It directly inhibits non-self-cells and promotes immunotherapy via T cells. The use of anti-CD47 mAb to blockade CD47-SIRPα is a potential therapeutic strategy in the deadly duo of COVID-19 and cancer. Early detection of other signaling pathways such as *anti*-MHC1 (or antiLILRB1) will regulate phagocytosis by macrophages.

The CD47-SIRPα interaction initiates a dephosphorylation cascade that has phosphotyrosines in myosin as a partial target. As a result, macrophage phagocytosis is restricted and the release of the “do not eat me” signal is facilitated. *In vivo*, this condition promotes immune tolerance in non-self-cells, which enables cancer or virus-infected cells to survive [11,12] (Fig. 1). The advantage of CD47 blocking is not limited to a single disease because CD47 is a host self-marker. The current data show that CD47 is upregulated in infected cells and uninfected DCs as a checkpoint response to infectious agent recognition or proinflammatory cytokines [13]. Since poxviruses and myxoma virus (M128L) are thought to be the best models for mimicking CD47 to spread the virus *in vivo*, we believe that CD47 overexpression can play an immunosuppressive role [14,15]. CD47 overexpression increases the number of SARS-CoV-2 infected cells that do not encode a CD47 mimic [16]. As a result, upregulation of CD47 in MQ cells may have consequences in the context of inflammation or infection. Wenzek et al. revealed that the expression of CD47 on immune cells seemed to disturb the antiviral immune response as CD47-deficient mice (CD47−/−) showed an augmented clearance of influenza A virus (IAV) [17].

### 1.2 CD47 and COVID-19 associated cancer

To the best of our knowledge, SARS-CoV-2-infected Caco-2 cells and Calu-3 cells had higher levels of CD47, which is known to facilitate immune escape in malignant and virus-infected cells. SARS-CoV-2 infection raises primary human monocyte levels of SIRPα, a CD47 binding partner. On the other hand, increased CD47 levels are associated with known risk factors such as advanced age and diabetes. Higher CD47 levels are associated with vascular disease, vasoconstriction, and hypertension, which may put those who have SARS-CoV-2 at risk for COVID-19-related consequences like pulmonary hypertension, pulmonary fibrosis, myocardial injury, stroke, and delayed acute kidney injury. As a result, CD47 expression caused by viruses and aging is a prospective mechanism that may contribute to severe COVID-19 and a potential target for antibody therapy [18]. It has been reported that cancer cells increase CD47 to prevent immune identification.

In the context of COVID-19 features observed in patients with cancer, are immune checkpoint inhibitors (ICIs) considered a friend or foe? ICI reverse immunosuppressive conditions by stimulating immune cells to recognize antigens. Anti-CD47 therapy may re-energize the exhausted MQ cells during viral infection, thereby suppressing SARS-CoV-2 spread. Furthermore, the overactivation of innate immune cells causes severe tissue damage and hyperinflammation, which exacerbates lung pathology in severe COVID-19 and cancer. In this case, ICI could be a foe [19]. Barkal et al. [20] demonstrated that disruption of the MHC-I axis by the inhibitory receptor LILRB1, an essential regulator of innate immune cells, can increase phagocytosis of tumor-associated macrophages *in vitro* or *in vivo*, and contribute to anti-signal-regulatory protein CD47 therapy. Thus, CD47 is critical for balancing the anti-inflammatory signals.

But given the COVID-19 pandemic, cancer patients have faced unprecedented complications. Cancer patients are an important group of patients that can help researchers understand the immune response to SARS-CoV-2 [19]. The topic of “cytokine storms” has been intriguing in infectious diseases and cancer during the COVID-19 pandemic. Uncontrolled monocyte and macrophage infections can result in cytokine storm syndrome in severe COVID-19 [6]. The emergence of a cytokine storm resulting in hyperinflammation is a common feature of cancer and severe COVID-19. In cancer patients, malignancy or anticancer treatments may impair the antiviral response to severe COVID-19 [21]. Several clinical trials have been conducted to investigate the efficacy of CD47 blockade in cancer patients [22,23], and previous findings [13,18,24] suggest that anti-CD47 therapy can affect viral infections, such as severe COVID-19. Therapy-induced innate immune effects (e.g., ICI) may present a unique opportunity to learn more about *anti*-SARS-CoV-2 regulators.

Previous paradigms have recognized that cancer patients are more vulnerable to severe COVID-19 complications and are referred to as “doubly unfortunate.” According to meta-analysis studies from China, Italy, and the United States, patients with colorectal cancer [25], hematological and breast cancers [26], and both lung cancer and COVID-19 [27] are more vulnerable to SARS-CoV-2 infection than other cancers because these malignancies are associated with hospitalization and high mortality rates. Lung cancer was the most prevalent type in COVID-19 patients, according to Hanafi et al. [28]. It can contribute to a negative prognosis because it is related to the largest risks of COVID-19 infection, death, and structural damage from lung cancer itself. They found that non-small cell lung cancer (NSCLC) (57.8%), which made up the majority of lung tumors (84.4%), was the most prevalent kind of other chest malignancy.

The clinical association between COVID-19 and cancer is based on studies of cytokines, IFN-I, and immune checkpoint signaling (e.g., CD47) [29]. Basic molecular insights can be translated into clinical tools to extend common therapeutic approaches against COVID-19 and related cancers.

Lung cancer is the most common type of cancer in the world. Immune checkpoint inhibitors that target the programmed cell death protein 1 (PD-1) axis can alter lung cancer treatment [30,31]. CD47 is overexpressed in many human cancers, including leukemia [32], lymphoma [33], multiple myeloma [31], and solid tumors such as breast [34], colon [35], hepatocellular carcinoma [36], and melanoma [31]. The expression of CD47 is linked to tumor invasion and metastasis. However, it is unclear how CD47 promotes metastasis in lung cancer. Although many recent studies have focused on the “do not eat me” signal through the interaction of CD47 with cancer or virus-infected cells and SIRPα with macrophages, other mechanisms may also contribute to the therapeutic effects of CD47. The small GTPase Cdc42 is a member of the Rho family and is overexpressed in a number of cancers. It is also a known metastasis regulator that is overexpressed in some human cancers, including lung cancer [37]. The activation of Cdc42 is caused by overexpression of CD47. Cdc42, a downstream signaling agent of CD47, is activated to promote neurite and filopodium formation [31,36]. In order to affect cellular metabolism, the interaction between CD47 and CDdc42, a downstream signaling factor, can promote the development of lamellipodia and filopodia protrusions [30].

In addition to cancer, there is growing evidence that disrupting CD47/SIRP signaling during infection increases macrophage-mediated phagocytosis [24]. In contrast to cancer, CD47 upregulation during infection results from endosomal stimulation of the PRR. Thus, activation of PRR signaling can improve immune responses by blocking the CD47 signaling pathway. The presence of inflammatory cytokines such as interferon-alpha or tumor necrosis factor-alpha (TNF-α) in the serum of patients with chronic infection explains why the host defense weakens during infection and cancer [38]. Cham et al. [13] recently discovered that CD47 expression is upregulated in early host responses to SARS-CoV-2 infection and that blocking CD47 with anti-CD47 therapy has a promising therapeutic effect during viral infection. Their findings showed that blocking CD47 improved APC function, and thus increased innate and adaptive immune responses. Therefore, anti-CD47 therapy can be widely used to treat viral diseases. The TNF-NFkB1 pathway regulates CD47 expression. Therefore, CD47 overexpression in cancer cells or SARS-CoV-2-infected cells is an essential survival strategy [38]. The role of infectious agents such as SARS-CoV-2 in forcing infected cells to overexpress CD47 to avoid immune monitoring remains unknown.

### 1.3 CD47 and SARS-CoV-2 infection

CD47 overexpression in tumors or SARS-CoV-2-infected cells is an ideal cell surface biomarker for immuno-oncology-based therapies. Blocking the CD47-SIRP interaction with the SARS-CoV-2 response is critical for cancer treatment and has the potential to be curative. The use of agents that block the CD47 and SIRP axis (antibodies targeting CD47 and SIRP, SIRP-Fc fusion proteins, and bi-specific antibodies) is a promising approach to modulate the immune system to reduce or prevent metastases and viral infections. As a result, anti-CD47 may specifically target MQ to mediate phagocytosis and clearance [39]. The CD47 signaling cascade is unknown in severe COVID-19 and related cancers. A better understanding of the CD47 signaling pathway opens new possibilities for anti-CD47 therapy in cancer patients, such as inhibiting tumor growth or SARS-CoV-2 replication in the early stages to prevent the development of severe COVID-19. Combination therapy with opsonizing antibodies, immunomodulators, and chemotherapeutic agents activates FcR on macrophages and stimulates a potent immune response. Mechanisms targeting the CD47-SIRP axis should inhibit the CD47/SIRP interaction while activating MQ (FcR) signaling [40].

CD47 has vasopressor activity, overexpression of CD47 inhibits the soluble cellular second messenger molecule (responsible for vasodilation) mediated by nitric oxide, resulting in hypertension in some people at a high risk of developing severe COVID-19. Loss of CD47 expression in older red blood cells results in the elimination of macrophage-mediated programmed cells [41]. In contrast, CD47 causes endothelial senescence with aging and poor vascular function through synergistic action with thrombospondin-1 (TSP1), a glycoprotein with numerous cellular functions including antiangiogenic activity, cell adhesion, cell-to-matrix adhesion, propagation, apoptosis, inflammation, and endothelial cell senescence [42,43]. The CD47-TSP1 link inhibits inflammasome, IL-1, and T-cell proliferation in APCs [44]. CD47 is an immunosuppressive molecule because of its role as a thrombospondin-1 receptor and its interaction with SIRP in immune cells [45]. The role of CD47 in the development of severe COVID-19 vascular pathology (including pulmonary hypertension, lung fibrosis, heart attack, stroke, acute renal injury, and prolonged COVID-19) is significant. Overexpression CD47 aids in the treatment of vascular diseases, vasoconstriction, and hypertension. As a result, people infected with SARS-CoV-2 are more likely to develop severe COVID-19 complications, such as pulmonary hypertension, pulmonary fibrosis, myocardial injury, stroke, and acute kidney injury. There is a link between the risk of diabetes and severe COVID-19 and the high expression of CD47. McLaughlin et al. [1] reported that the levels of CD47 were increased in SARS-CoV-2-infected Calu-3 cells, Caco-2 cells, and air-liquid interface cultures of primary human bronchial epithelial cells. Consequently, CD47 expression has complex biological consequences. Thus, the overexpression of CD47 explains why immunocompromised individuals are more susceptible to severe COVID-19.

## 2 Conclusion

The clinical efficacy of therapeutic antibodies that target T-cell checkpoint molecules has led to a focus in clinical oncology studies on combination therapy utilizing checkpoint inhibitors in addition to other medications. Interesting therapy options, in this case, include those that inhibit the CD47/SIRP pathway. Cancer therapy should focus on CD47, which is overexpressed in tumor cells. Drugs that target the CD47/SIRP axis should promote macrophage signaling in addition to inhibiting the CD47/SIRP interaction. Both immune checkpoint inhibitors and opsonizing antibody combinations in therapy show promise.

Finally, knocking down or blocking CD47 as an immunosuppressive protein increases the innate and adaptive immune responses. Anti-CD47 therapy boosts the phagocytic activity of virus-infected cells, antigen processing by antigen-presenting cells (APCs), and the activation capacity of antiviral CD8^+^ T cells. Anti-CD47 therapy has emerged as a novel target for cancers associated with emerging infections, such as SARS-CoV-2. The mAb-mediated CD47 blockade is currently being studied for cancer treatment and has the potential to be developed and designed as an immunotherapeutic target for viral diseases. Because increased CD47 levels have been linked to SARS-CoV-2 infection and CD47 can be used as a drug target, we propose that combining mAb-mediated CD47 blockade with ICI therapy in COVID-19-infected cancer patients could promote macrophage-mediated immunotherapy.

## Ethics approval

Not applicable.

## Consent to participate

Not applicable.

## Consent to publish

Not applicable.

## Author contribution statement

All authors listed have significantly contributed to the development and the writing of this article.

## Data availability statement

No data was used for the research described in the article.

## Additional information

No additional information is available for this paper.

## Declaration of competing interest

The authors declare that they have no known competing financial interests or personal relationships that could have appeared to influence the work reported in this paper.

## Abbreviation

MQ: Macrophages
ICI: Immune checkpoint inhibitors
CD47: Cluster of differentiation 47
SIRPα: Signal regulatory protein α
DCs: Dendritic cells
CTLs: Cytolytic T lymphocytes
ITIM motif: Immunoreceptor tyrosine-based inhibitory motif
SHP1/2: Src homology phosphate 1/2
SARS-CoV-2: severe acute respiratory syndrome coronavirus 2
COVID-19: Coronavirus disease 2019
MHC-I: Major histocompatibility complex class I
LILRB1: Leukocyte immunoglobulin like receptor B1
PD-1: Programmed cell death protein 1
Cdc42: Cell division control protein 42
FcR: Fragment crystallizable receptor
FcγR: Fc gamma receptor
IFN-I: type I interferon
IL-1b: Interleukin 1b
PRR: Pattern recognition receptor
NO: Nitric oxide
TSP1: Thrombospondin-1 receptor
APC: Antigen presenting cell
mAb: monoclonal antibody
